# Editor's note: Chimpanzee vowel-like sounds and voice quality suggest formant space expansion through the hominoid lineage

**DOI:** 10.1098/rstb.2023.0201

**Published:** 2023-07-31

**Authors:** Philosophical Transactions B Editorial team

*Phil. Trans. R. Soc. B* **377**, 20200455. (Published online 15 November 2021) (https://doi.org/10.1098/rstb.2020.0455)

This is an update to the Expression of concern (Published online 26 December 2022): https://doi.org/10.1098/rstb.2022.0476 [1].

The journal's editorial team wish to update readers on this investigation which is ongoing.

The authors acknowledge that there are problems with the analyses in this paper, and they are currently re-analysing the data. The response will be independently reviewed and either a correction or retraction will be published in due course.
